# An investigation into the impact of enteric coated of aspirin in patients with newly diagnosed ischemic stroke (ECASIS)

**DOI:** 10.1097/MD.0000000000020307

**Published:** 2020-05-15

**Authors:** Mohamed Nabil Elshafei, Yahia Imam, Mouhand F.H. Mohamed, Arwa Ebrahim AlSaud, Mohamed Sayed Ahmed, Khaldun Obeidat, Razan Saeid, Mohamed Ali, Ibtihal M. Abdallah, Aeijaz Sultan Parray, Mohammed Ibn-Masoud Danjuma

**Affiliations:** a Clinical Pharmacy Department; b Neurology Department, Hamad General Hospital, Hamad Medical Corporation; c Weill Cornell Medicine-Qatar; d Internal Medicine Department, Hamad General Hospital; e Interim-Lab, Hamad Medical City, Hamad Medical Corporation; f Qatar University, College of Medicine, Doha, Qatar.

**Keywords:** aspirin, enteric coated, ischemic, plain, stroke, thromboxane B2, TXB2

## Abstract

**Introduction:**

Uncertainty remains regarding the impact of enteric-coated (EC) aspirin as it relates to the reduction of cardiovascular risk. We hypothesize that EC formulation based on a previous report may blunt aspirin response as evidenced by reduced Thromboxane A2 (TXA 2) levels in diabetic patients. Thus, it was imperative to ascertain and validate the effect of the EC formulation of Aspirin on the Thromboxane B2 (TXB2) level.

**Methods/design:**

An open-label consecutive randomized interventional controlled trial. Patients with newly diagnosed ischemic stroke who are just about to start Aspirin were assessed for eligibility and inclusion in our trial. Consecutive patients (admitted to the stroke unit of Hamad General Hospital, Hamad Medical Corporation, Doha, Qatar) will be randomized to receive either EC aspirin or plain Aspirin. They will be required to continue taking them throughout the study (3 days). Demographics and laboratory records of the study participants will be abstracted from online records. Further study variables will be obtained manually in designated case record forms (CRF). The primary outcomes are the incidence of aspirin non-responders (level of residual serum TXB2 associated with elevated thrombotic risk (<99.0% inhibition or TXB2 >3.1 ng/mL) within 72 h after three daily aspirin doses). Whereas secondary outcomes are the incidence of GIT bleeding of various preparations of Aspirin. The study was approved by MRC and IRB of Hamad Medical Corporation (**MRC number: 01-18-156).**

**Discussion:**

This trial will determine potential differences in the efficacy of EC Aspirin and plain Aspirin on the Thromboxane B2 level. Additionally, it will ascertain the tolerability and safety of both formulations of Aspirin in patients with newly diagnosed ischemic stroke. These results will either support the current notion of no difference between the two formulations. However, if a difference is found, this will invite for future trials exploring clinical outcomes occurrence between various formulations.

**Clinical trial registration:**

Clinicaltrials.gov NCT04330872 registered on April 2, 2020

## Abstract 2

Supplemental Digital Content is available in the text

## 1 Introduction

The safety and efficacy of antiplatelet therapy in secondary prevention of wide-ranging cardiovascular risk (coronary artery disease, stroke-related outcomes, and peripheral vascular disease) have been well established from some systematic studies and meta-analysis^[1,2,3,4,5,6,7,8,9]^ This has resulted in their incorporation into most national and international treatment guidelines.^[10,11,12]^ Recently, concerns have been raised regarding the potential of attenuation of aspirin pharmacokinetic (PK) effects following administration in an EC formulation.^[13,14,15,16,17,18]^ The recurrence of stroke and ischemic heart disease (IHD)-related outcomes have been a subject of concern over the past few years. Anti-platelet therapy resistance has been a particular subject of such concern.^[19,20,21]^ Aspirin resistance whilst secondary to several factors appears in recent reports to be influenced by amongst others its enteric coating. Various systematic and mechanistic studies have explored the effect of enteric coating on aspirin effectiveness.^[13,14,16,17,18]^ Whilst the outcomes of these reports have not been uniform or unanimous, it raises considerable concern regarding the propensity of enteric coating to significantly attenuates its level and reduce its effectiveness in primary and secondary prevention. In a seminal mechanistic report, showed significantly low TXB2 level (a surrogate marker of aspirin response) in obese type 2 DM patients on EC Aspirin compared to their sex and age-matched cohorts on plain aspirin.^[16]^

Despite the evident limitation of this report (been a proof of concept study with a mechanistic design), uncertainty remains regarding the impact of this on long-term hard cardiovascular outcomes. In this study, we aim to firstly validate this reported association in a population of stroke patients, and in subsequent prospective studies ascertain the effect of enteric coating of aspirin on hard clinical endpoints such as the major adverse cardiovascular events (MACE).

## 2 Methods/design

### 2.1 Study design and settings

**Figure 1:**
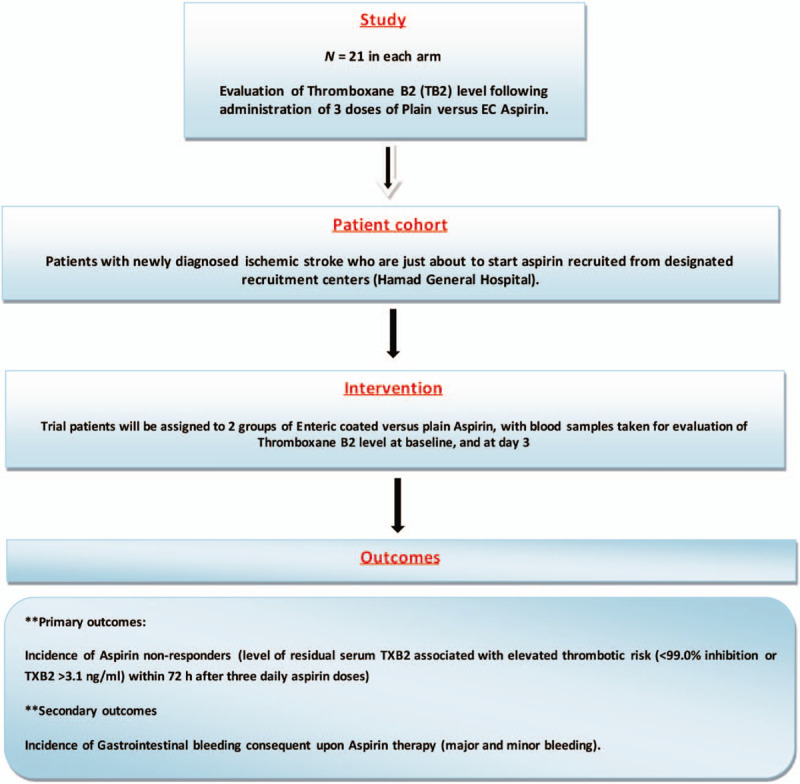
Flow chart depicting the study procedures.

This is an open-label, two-arms, parallel, non-randomized (consecutive patients’ randomization) proof of concept, phase 4 pharmacokinetic/ pharmacodynamic study. We will recruit eligible patients from the stroke unit of designated recruitment centers (Hamad General Hospital). Patients who satisfy pre-specified inclusion criteria will be invited to participate in the study. Patients who agreed to take part will be screened, counseled, and enrolled in the study. They will be required to sign a consent form before allocation. This study was approved by the local MRC and The Institutional Review Board (IRB) (Hamad Medical Corporation, **MRC number: 01–18–156**) and registered in clinicaltrials.gov (NCT04330872) (Fig. 1).

### 2.2 Setting and participants

Patients (male and female) (N = 42) with newly diagnosed ischemic stroke who are just to start aspirin therapy, and who attend stroke units of designated recruitment centers (Hamad General Hospital). We will enroll participants meeting the following inclusion criteria:

#### 2.2.1 Inclusion criteria

1. Newly diagnosed ischemic stroke who are just about to start Aspirin.
2. No prior history of cardiovascular morbidity (including IHD, chronic kidney disease, peripheral vascular disease)
3. 18 to 75 years.

#### 2.2.2 Exclusion criteria

1. Concomitant antiplatelet therapy (irrespective of the duration of the treatment).
2. Patients on any prostaglandins-related medications (non-steroidal anti-inflammatory drugs, misoprostol, and other ant secretory drugs among others).
3. Any salicylate-containing supplements.
4. Patients on the NG tube will be excluded from the study.

### 2.3 Randomization, allocation concealment, and blinding

Consecutive patients meeting the eligibility criteria will be explained about the study procedures by one of the trial investigators following that that patient will be consented by designated study investigators (Supplementary materials 1. Consent form). If the patient consents to participate in our trial, then he/she will be allocated to either plain or EC aspirin arms following their admission order. Outcome assessors and data analysts are blinded to patient allocation.

### 2.4 Interventions

Once allocated to one of the two study arms, the study participants will be administered the study medication as either enteric-coated or plain Aspirin on day 1. Patients assigned will be prescribed plain loading dose (Dispersible 300 mg stat followed by Aspirin 75 mg tablets, Actavis UK Ltd) or EC Aspirin loading dose (300 mg stat followed by100 mg, Bayer, Germany) for 3 days (study duration). Irrespective of the design of the study, all patients enrolled in the study will receive other standards of care management according to HMC ischemic stroke guidelines. Patients will be required to give blood samples (10 mL) for estimation of the TXB2 level at baseline and on day 3, which marks the end of the study. Blood samples will be taken as part of the patient's routine investigation/care by the phlebotomist. Study samples will be taken by a designated study investigator as per standard protocols consistent with HMC guidelines. Study samples will be kept in labeled with anonymous patient-specific identifiers and kept in study refrigerators until completely used. Patients will be kept on other treatments constituting the usual standards of care management for established ischemic stroke, and this will be maintained throughout the study. We anticipate that it would take about one month to complete patient recruitment. Following the end of the study, laboratory analysis of biological samples will be carried out within 3 weeks with subsequent data evaluation and analysis.

### 2.5 Data collection

Data will be collected in case records form (Supplementary materials 2). Data will be transferred to electronic records and stored in the secure computers of Hamad Medical Corporation. The access log to these computers will be restricted to the primary investigator. Case record forms will be kept at safety cabinets in HMC. When there is a need for data alteration in the case record form, this will involve striking through the data with a straight line and countersigned by the investigator involved. Patients have been educated that they can withdraw from the study at any time, and when such a decision is made, patient's records (both papers and electronics) will be removed as well as blood samples and destroyed in keeping with regulatory requirements. Additionally, the parent medical teams will be notified.

### 2.6 Primary outcomes

Incidence of Aspirin non-responders at day 3 (level of residual serum TXB2 associated with elevated thrombotic risk (<99.0% inhibition or TXB2 >3.1 ng/mL).

### 2.7 Secondary outcomes

Incidence of Gastrointestinal bleeding consequent upon aspirin therapy (major and minor bleeding).

Gastrointestinal bleeding is classified as major bleeding: 2 g loss of hemoglobin or requirement of hospitalization; Minor bleeding: any bleeding less severe than above.^[22]^

### 2.8 Statistical consideration

#### 2.8.1 Sample size

There are no previous studies to allow for a robust estimation of the study sample size. However, given that this is proof of concept study, we estimated that a sample size of 21 patients in each arm of the study would yield an approximate 80% power to detect 50% difference in population mean, for two-sided significance and an alpha level of 0.05.

#### 2.8.2 Analysis set

The full analysis Set utilizing Intention to treat methodology will be used for the primary efficacy analysis. Per-protocol set analysis will be attempted for the primary efficacy outcomes.

#### 2.8.3 Statistical analysis

Descriptive statistics would be carried out to summarize the demographic, clinical, and laboratory characteristics of the study population. Within and between-group, changes in study parameters will be analyzed using Wilcoxon signed Rank test or Student's *t* test as appropriate. Median change of TXB2 levels from baseline will be determined by repeated measures analysis (ANOVA). Spearman correlation coefficients would be used to estimate the relationship between independent variables. Variables with *P* < .2 in univariate analyses will be entered into a multivariate model. Multivariate linear regression analysis will be used to assess the effects of covariates on thromboxane A2 levels. All analyses will be carried with StatsDirect version 2.7.9 (StatsDirect Ltd, Altrincham, Cheshire, UK).

### 2.9 Quality control and trial management

Study participants will be reassured about the maintenance of usual standards of care for the various CV risk and morbidities. Where any serious adverse event of note came to the attention of the study investigator, this information will be made available to the primary team superintending the patient's care. Additionally, Aspirin has had quite extensive use across a broad range of CV risks and morbidities and is the core of stroke management; therefore, the data monitored committee (DMC) was not deemed necessary by the IRB in our study. Data will be stored for at least 5 years after which it will be destroyed/ deleted. The study has been conducted after review and approval from MRC and IRB.

## 3 Discussion

Our study is the third study assessing the possible effect of enteric-coated aspirin formulation on TXB2 inhibition. The first study was in 2006 by Cox et al. In their study of seventy-one healthy volunteers taking both Plain and EC aspirin in a randomized crossover design, they concluded that EC aspirin was not as effective as plain Aspirin in terms of TXB2 inhibition. The second study was in 2017 by Bhatt et al. It examined 40 diabetic patients in a randomized crossover design. Again, it concluded the inferiority of EC aspirin formulations compared to plain Aspirin (High proportion of incomplete TXB2 inhibition).

The findings from the previously mentioned trials prompted us to perform the first trial (up to the best of our knowledge), examining the level of TXB2 inhibition in stroke patients taking various formulations of Aspirin (plain vs EC aspirin). If a difference is TXB2 is found in our trial, this significant finding will invite additional clinical trials exploring the efficacy of various formulations of Aspirin measured via hard clinical outcomes such as major adverse cardiovascular events (MACE).

## Acknowledgments

We would like to acknowledge the Qatar National Library for funding the open-access publication of this paper.

## Author contributions

MNE conceived the research idea. MNE and MID designed the initial study protocol submitted to MRC. MID is responsible for the statistical design and analysis. MNE, MID, and MFHM wrote the initial draft of this paper and revised the final version and approved it for submission. All other authors (MSA, AEA, KO, RA, MA, IMA, and ASP) critically reviewed the initial draft and approved the final version for publication. All authors carefully read and approved the final version of the manuscript.

## Supplementary Material

Supplemental Digital Content

## Supplementary Material

Supplemental Digital Content
