# Supplementary material for: An investigation into the impact of enteric coated of aspirin in patients with newly diagnosed ischemic stroke (ECASIS)
Source: Medicine (Baltimore). 2020 May 15;99(20):e20307. doi: 10.1097/MD.0000000000020307 (PMC7254488; doi:10.1097/MD.0000000000020307)
Supplement: Supplemental Digital Content [file medi-99-e20307-s001.docx]

| 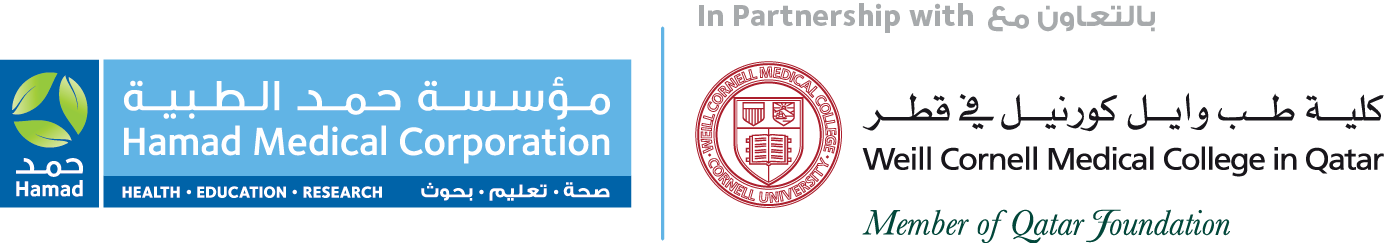 |
| --- |
| **1. Title of research** |
| An Investigation Into The Impact Of Enteric Coated Of Aspirin In Patients With Newly Diagnosed Ischemic Stroke. |
| **2. Principal Investigator** |
| Dr . Mohamed Nabil Hamad General Hospital |
| **3. Why are we inviting you to join this research?** |
| The investigator and colleagues at Hamad Medical Corporation (HMC) [Hamad General Hospital] are conducting this research.  We are inviting you to join because you have been diagnosed by ischemic stroke and you will start taking aspirin which will make you eligible for this study. |
| **4. What should you know about this research?** |
| - We will explain the research to you - Whether or not you join is your decision (you can accept or refuse no matter who is inviting you to participate) - Please feel free to ask questions or mention concerns before deciding, or during or after the research - You can say yes but change your mind later - We will not hold your decision against you |
| **5. Who can you talk to?** |
| If you have questions or concerns, or if you think the research has hurt you, talk to the research team at:  [Dr. Mohammed Danjuma <Tel:55896229> , Dr.Mohamed Nabil <Tel:30529249> , Dr.Yehia Imam Tel: 55246887 ]  If you have questions about your rights as a volunteer, or you want to talk to someone outside the research team, please contact:   - HMC Institutional Review Board (HMC-IRB) Chair at 5554 6316 - HMC-IRB Office at 4025 6410 (from Sunday to Thursday between 7:00am-3:00pm) or email at [irb@hamad.qa](mailto:irb@hamad.qa) |
| **6. Why are we doing the research?** |
| Amongst people who have had stroke and need aspirin as part of their treatment, we are not definitely sure at the moment if giving the aspirin in a coated form affects its ability to work in the body. Our initial assessment from what we know from previous research in patients with Diabetes is that coating the aspirin may reduce its ability to work by preventing further strokes.  In this study, we aim to firstly investigate if coating aspirin reduces its ability to reduce the amount of certain chemical called Thromboxane B2, which has been used as a measure of how well aspirin works. |
| **7. How long will the research take?** |
| We think that you will be in the research for three days.  We expect the research to last for 2 years during which we would have recruited to total number of patients we need for the study and follow up on other study measurements. The research will last for 2 years. |
| **8. How many people will take part?** |
| We plan to recruit 42 study participants. |
| **9. What happens if you take part?** |
| If you agree to join, we will ask you to do the following:   1. You will start taking either enteric coated aspirin or plain aspirin for 3 days and beyond as part of standard of care for treatment of ischemic stroke. 2. You will be required to give one blood sample (10 mls) on day 3. 3. You will be randomized into either of the 2 study arms. Randomization means that you are put into a group by chance. It is like flipping a coin. Neither you nor the researchers choose which group you will be in. You will have a one in two chance of being place in a specific group.   4- Data will be collected from Medical Records. |
| **10. Could the research be bad for you?** |
| It is a policy of this study recruitment centre (HMC) as part of patients’ usual standards of care to prescribe either plain or EC aspirin without any distinct individual difference. Therefore by allocating study population into EC or plain aspirin arm, we don’t anticipate any additional risk to study participants.  Common adverse effects of both two formulations of aspirin (Frequency Not Defined):   - Angioedema - Bronchospasm - CNS alteration - Dermatologic problems - GI pain, ulceration, bleeding - Hepatotoxicity - Hearing loss - Nausea - Platelet aggregation inhibition - Premature hemolysis - Rash - Renal damage - Tinnitus - Urticaria - Vomiting |
| **11. Could the research be good for you?** |
| There are no benefits to you from joining this research. However, possible benefits to others include knowing whether there is difference between the two formulations of aspirin, subsequently by doing further studies could guide us to whether there is difference to use one of them better than another in ischemic stroke. |
| **12. What happens to information about you?** |
| Data will be collected in case records form. Data will be transferred to electronic records and stored in the secure computers of Hamad Medical Corporation. The access log to these computers will be restricted to PI, COI and, other study personnel.  Case record forms will be kept at safety cabinets in HMC. When there is need for data alteration in the case record form, this will involve striking through the data with straight line and countersigned by the investigator involved.  “We cannot guarantee complete secrecy, but we will limit access to information about you. Only people who have a need to review information will have access. These people might include:   - Members of the research team and or representatives whose work is related to the research or to protecting your rights and safety. - Representatives of the Qatar Supreme Council of Health and medical research center who make sure the study is done properly and that your rights and safety are protected - Your doctors and nurses |
| **13. What if you don’t want to join?** |
| You can say no and we will not hold it against you. |
| **14. What if you join but change your mind?** |
| You can stop participating at any time and we will not hold it against you. The information and samples we have already collected about you will be removed and destroyed in line with regulatory procedures. |
| **15. What else should you know?** |
| This research is funded by Hamad Medical Corporation. |

| Signature Page for Capable Adult |
| --- |
| Volunteer |
| *I voluntarily agree to join the research described in this form.*  Printed Name of Volunteer  Signature of Volunteer Date |
| Person Obtaining Consent |
| *I document that:*   - *I (or another member of the research team) have fully explained this research to the volunteer.* - *I have personally evaluated the volunteer’s understanding of the research and obtained their voluntary agreement.*   Printed Name of Person Obtaining Consent  Signature of Person Date  Obtaining Consent |
| Witness (if applicable) |
| *I document that the information in this form (and any other written information) was accurately explained to the volunteer, who appears to have understood and freely given Consent to join the research.*  Printed Name of Witness  Signature of Witness Date |
