# Supplementary material for: An investigation into the impact of enteric coated of aspirin in patients with newly diagnosed ischemic stroke (ECASIS)
Source: Medicine (Baltimore). 2020 May 15;99(20):e20307. doi: 10.1097/MD.0000000000020307 (PMC7254488; doi:10.1097/MD.0000000000020307)
Supplement: Supplemental Digital Content [file medi-99-e20307-s002.docx]

| **Study Title:** An Investigation Into The Impact Of Enteric Coated Of Aspirin In Patients With newly diagnosed Ischemic Stroke | **CASE REPORT FORM** |
| --- | --- |
| **Study number: MRC-01-18-156** |  |
| **Patient ID:** \|__\|__\| / \|__\|__\|__\| |  |

**Study Title:**

An Investigation Into The Impact Of Enteric Coated Of Aspirin In Patients With newly diagnosed Ischemic Stroke

**Study number:**

**MRC-01-18-156**

**General Guidelines**

▪ Entries in the CRF to be made using preferably Black ink ballpoint pen

▪ Ensure all entries are accurate, legible to others and verifiable with the source data

▪ In case of correction:

- Do not overwrite or erase!
- Do not use correction materials (whiteners, cello tapes, and bleaching)
- Write the correct entry nearby
- Dare and initial the correction (see example given below)


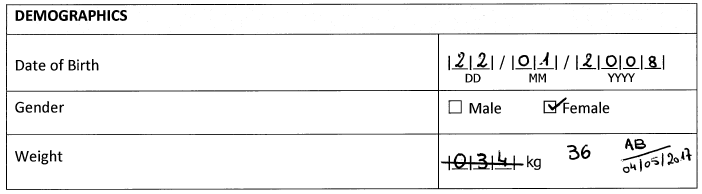


▪ Acceptable abbreviations are the following:

- Unknown: UNK
- Not Done: ND
- Not Applicable: NA

▪ In questions where some of the possible options listed has/have to be selected, it should be ticked (as shown below)


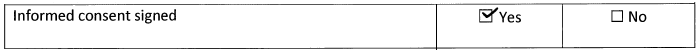


| **Study Title: An Investigation Into The Impact Of Enteric Coated Of Aspirin In Patients With newly diagnosed Ischemic Stroke** | **ELIGIBILITY** |
| --- | --- |
| **Study number: MRC-01-18-156** |  |
| **Patient ID:** \|__\|__\| / \|__\|__\|__\| |  |

| **INCLUSION CRITERIA (all criteria must be answered YES for subject to be eligible**) | | | |
| --- | --- | --- | --- |
| 1 | Newly diagnosed ischemic stroke who are just about to start aspirin. | ☐ Yes | ☐ No |
| 2 | No prior history of cardiovascular morbidity (including ischemic heart disease, chronic kidney disease, peripheral vascular disease) | ☐ Yes | ☐ No |
| 3 | 18- 75 years | ☐ Yes | ☐ No |
| 4 | Patients with ischemic stroke who underwent reperfusion intervention (catheter directed thrombolysis and/or thrombectomy) regardless of the time of presentation (i.e. within or outside the therapeutic window as the managing stroke deem fit. | ☐ Yes | ☐ No |

| **EXCLUSION CRITERIA (all criteria must be answered NO for subject to be eligible)** | | | |
| --- | --- | --- | --- |
| 1 | Concomitant anti-platelet therapy (irrespective of the duration of the treatment). | ☐ Yes | ☐ No |
| 2 | Patients on any prostaglandins related medications (non steroidal anti-inflammatory drugs, misoprostol, and other antisecretory drugs among others). | ☐ Yes | ☐ No |
| 3 | Any salicylate containing supplements | ☐ Yes | ☐ No |
| 4 | Patients with NG tube | ☐ Yes | ☐ No |

| **Continuation of patient in the trial** |
| --- |

| The patient fulfills the necessary criteria and is deemed fit to continue in the trial and is able to perform all follow-up exams. | ☐ Yes | ☐ No |
| --- | --- | --- |

| **Study Title:** An Investigation Into The Impact Of Enteric Coated Of Aspirin In Patients With newly diagnosed Ischemic Stroke | **BASELINE** |
| --- | --- |
| **Study number: MRC-01-18-156** |  |
| **Patient ID:** \|__\|__\| / \|__\|__\|__\| |  |

| **INFORMED CONSENT** | | |
| --- | --- | --- |
| Informed consent signed | ☐ Yes | ☐ No |
| Date informed consent signed | \|__\|__\| / \|__\|__\| / \|__\|__\|__\|__\|  DD MM YYYY | |
| Time informed consent signed | \|__\|__\| : \|__\|__\| ☐ am ☐ pm  HH MM | |

| **DEMOGRAPHICS** | |
| --- | --- |
| Date of Birth | \|__\|__\| / \|__\|__\| / \|__\|__\|__\|__\|  DD MM YYYY |
| Gender | ☐ Male ☐ Female |
| Weight | \|__\|__\|__\| kg |

| **PAST HISTORY** | | |
| --- | --- | --- |
|  | ☐ Yes | ☐ No |
|  | \|__\|__\|__\|  \|__\|__\|__\| | |
|  | ☐ Yes | ☐ No |
|  | ☐ Yes | ☐ No |
|  | ☐ Yes | ☐ No |

| **Study Title:** An Investigation Into The Impact Of Enteric Coated Of Aspirin In Patients With newly diagnosed Ischemic Stroke | **BASELINE (laboratory data)** |
| --- | --- |
| **Study number: MRC-01-18-156** |  |
| **Patient ID:** \|__\|__\| / \|__\|__\|__\| |  |

| **Variables** | | |
| --- | --- | --- |
| Creatinine | ☐ At screening | Day 3 |
| Urea |  |  |
| PT |  |  |
| APTT |  |  |
| Thromboxane B2 |  |  |
| Platelet |  |  |
| Hemoglobin |  |  |

| **Study Title:** An Investigation Into The Impact Of Enteric Coated Of Aspirin In Patients With newly diagnosed Ischemic Stroke | **EVALUATION** |
| --- | --- |
| **Study number: MRC-01-18-156** |  |
| **Patient ID:** \|__\|__\| / \|__\|__\|__\| |  |

| **TREATMENT** | | | |
| --- | --- | --- | --- |
|  | | ☐ Yes | ☐ No |
|  | | ☐ Treatment Medicine  ☐ Other, indicate: _____________ | |
| Date: \|__\|__\| / \|__\|__\| / \|__\|__\|__\|__\|  DD MM YYYY | Time: \|__\|__\| : \|__\|__\| ☐ am ☐ pm  HH MM | | |

| **RANDOMIZATION** | | |
| --- | --- | --- |
| Has patient been randomized? | ☐ Yes | ☐ No |
| Randomization number: | \|__\|__\|__\|__\|__\|__\| | |

| **Study Title:** An Investigation Into The Impact Of Enteric Coated Of Aspirin In Patients With newly diagnosed Ischemic Stroke | **INTERVENTION** |
| --- | --- |
| **Study number: MRC-01-18-156** |  |
| **Patient ID:** \|__\|__\| / \|__\|__\|__\| |  |

| **INTERVENTION** | | | |
| --- | --- | --- | --- |
| Did patient receive the study intervention (name of study drug)? | | ☐ Yes | ☐ No |
| Date: \|__\|__\| / \|__\|__\| / \|__\|__\|__\|__\|  DD MM YYYY | Time: \|__\|__\| : \|__\|__\| ☐ am ☐ pm  HH MM | | |

| **Study Title:** An Investigation Into The Impact Of Enteric Coated Of Aspirin In Patients With newly diagnosed Ischemic Stroke | **ADVERSE EVENT** |
| --- | --- |
| **Study number: MRC-01-18-156** |  |
| **Patient ID:** \|__\|__\| / \|__\|__\|__\| |  |

Has the patient experienced any Adverse Event since enrollment in the study? ☐ Yes ☐ No

If Yes, specify below:

|  | **Adverse event description** | **Start Date** | **End Date** | **Severity**  1= Mild  2= Moderate  3= Severe | **In case of SAE- Please specify the criteria**  1= Death  2= Life threatening  3= Hospitalisation or prolongation of existing hospitalisation  4= Persistent or significant disability/incapacity  5= Congenital abnormality/birth defect | **Causality assessment**  1= Certain  2 = Probable/  Likely  3= Possible  Unlikely  4= Conditional/  Unclassified  5= Assessable/  Unclassifiable | **Action taken**  **with trial treatment**  1= Dose modification  2= Discontinuation of the IMP  3= Not applicable  4= Treatment continued without change | **Outcome**  1= Resolved  2= Resolved with sequelae  3= Ongoing  4= Fatal  5= Unknown |
| --- | --- | --- | --- | --- | --- | --- | --- | --- |
| 1. | **Dyspeptic symptoms** | \|__\|__\| / \|__\|__\| / \|__\|__\|__\|__\|  DD MM YYYY | \|__\|__\| / \|__\|__\| / \|__\|__\|__\|__\|  DD MM YYYY |  |  |  |  |  |
| 2. | **Minor hemorrhage^1^** | \|__\|__\| / \|__\|__\| / \|__\|__\|__\|__\|  DD MM YYYY | \|__\|__\| / \|__\|__\| / \|__\|__\|__\|__\|  DD MM YYYY |  |  |  |  |  |
| 3. | **Major hemorrhage^1^** | \|__\|__\| / \|__\|__\| / \|__\|__\|__\|__\|  DD MM YYYY | \|__\|__\| / \|__\|__\| / \|__\|__\|__\|__\|  DD MM YYYY |  |  |  |  |  |
| 4. |  | \|__\|__\| / \|__\|__\| / \|__\|__\|__\|__\|  DD MM YYYY | \|__\|__\| / \|__\|__\| / \|__\|__\|__\|__\|  DD MM YYYY |  |  |  |  |  |
| 5. |  | \|__\|__\| / \|__\|__\| / \|__\|__\|__\|__\|  DD MM YYYY | \|__\|__\| / \|__\|__\| / \|__\|__\|__\|__\|  DD MM YYYY |  |  |  |  |  |
| 6. |  | \|__\|__\| / \|__\|__\| / \|__\|__\|__\|__\|  DD MM YYYY | \|__\|__\| / \|__\|__\| / \|__\|__\|__\|__\|  DD MM YYYY |  |  |  |  |  |
| 7. |  | \|__\|__\| / \|__\|__\| / \|__\|__\|__\|__\|  DD MM YYYY | \|__\|__\| / \|__\|__\| / \|__\|__\|__\|__\|  DD MM YYYY |  |  |  |  |  |
| 8. |  | \|__\|__\| / \|__\|__\| / \|__\|__\|__\|__\|  DD MM YYYY | \|__\|__\| / \|__\|__\| / \|__\|__\|__\|__\|  DD MM YYYY |  |  |  |  |  |

**1. As per international guidelines defined those terms.**

| **Study Title: :** An Investigation Into The Impact Of Enteric Coated Of Aspirin In Patients With newly diagnosed Ischemic Stroke | **PI SIGNATURE** |
| --- | --- |
| **Study number: MRC-01-18-156** |  |
| **Patient ID:** \|__\|__\| / \|__\|__\|__\| |  |

I confirm that I have reviewed all the data in this Case Report Form and verify that it is a true and accurate reflection of this patient’s participation in the Clinical Investigation.

| Principal Investigator signature |  |
| --- | --- |
| Signature date | \|__\|__\| / \|__\|__\| / \|__\|__\|__\|__\|  DD MM YYYY |
